# Sex Differences in Statin Intolerance: Insights From the CLEAR Outcomes Trial

**DOI:** 10.1002/clc.70288

**Published:** 2026-04-01

**Authors:** Sahar Naderi, Danielle Brennan, Na Li, A. Michael Lincoff, Stephen J. Nicholls, Steven E. Nissen, Jorge Plutzky, Heather A. Powell, Paula Robinson, Leslie Cho

**Affiliations:** ^1^ Division of Cardiology, Kaiser Permanente Northern California Oakland California USA; ^2^ Cleveland Clinic Coordinating Center for Clinical Research Cleveland Ohio USA; ^3^ Esperion Therapeutics Inc. Ann Arbor Michigan USA; ^4^ Victorian Heart Institute Monash University Melbourne Victoria Australia; ^5^ Cleveland Clinic Cleveland Ohio USA; ^6^ Brigham and Women's Hospital Boston Massachusetts USA

**Keywords:** adverse events, bempedoic acid, low‐density lipoprotein cholesterol, sex, statin intolerance, tolerability

## Abstract

**Introduction:**

The CLEAR Outcomes study showed that bempedoic acid reduced LDL cholesterol (LDL‐C) and cardiovascular risk in patients with statin intolerance. CLEAR Outcomes included 48% female participants.

**Objective:**

To describe the differences in baseline clinical characteristics and tolerability of bempedoic acid by sex.

**Methods:**

Descriptive analysis of data from a double‐blind, randomized, placebo‐controlled cardiovascular outcome study in which 13 970 primary or secondary prevention patients at high cardiovascular risk with statin intolerance and baseline LDL‐C ≥ 100 mg/dL were randomized to bempedoic acid 180 mg daily or placebo.

**Results:**

At baseline, females (48%) were older, with less use of lipid lowering therapies, and more often without an ASCVD history. Females had more peripheral and cerebrovascular disease and less coronary artery disease. The incidence of myalgia was 5.4% versus 5.9% for females and 5.8% vs 7.6% for males treated with bempedoic acid versus placebo, respectively. Treatment discontinuation rates were lower with bempedoic acid than placebo in females (30.5% vs 33.4%) and males (27.9% vs 30.0%). Bempedoic acid discontinuation because of myalgia was comparable between females (1.8% vs. 1.6%) and males (1.7% vs 2.1%) versus placebo, respectively.

**Conclusions:**

This is the only cardiovascular outcomes trial to date that exclusively enrolled patients with statin intolerance, and the largest contemporary study of lipid lowering therapy in females, a group historically underrepresented in clinical trials. Bempedoic acid was equally tolerated by both sexes and represents a viable alternative to statins to reduce cardiovascular risk.

**Trial Registration:**

ClinicalTrials.gov identifier: NCT02993406.

## Graphical abstract

The global CLEAR Outcomes Trial was notable for enrolling 48% females. The study data set offers a unique opportunity to identify any differences in demographics, clinical characteristics, and tolerability of treatment with bempedoic acid between females and males with statin intolerance. While some differences in baseline characteristics among males and females were observed, bempedoic acid was equally tolerated by both sexes.

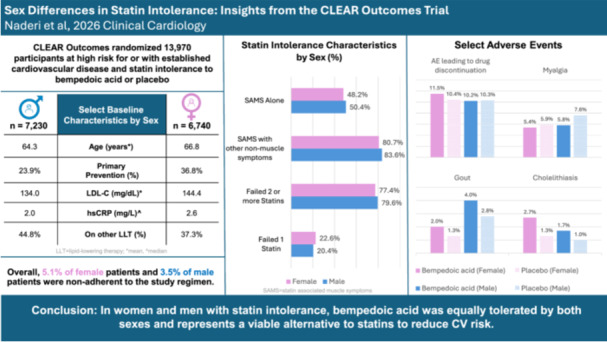

## 1 Introduction

Statin therapy to lower low‐density lipoprotein cholesterol (LDL‐C) is established as a fundamental component of reducing cardiovascular events in patients with known cardiovascular disease or those deemed high risk for such events [1]. Despite their demonstrated benefits, multiple reports reveal significant barriers exist with adherence to statin use due to side effects, with 7% to 29% of patients reporting adverse musculoskeletal effects that prohibit or limit statin use [2, 3, 4]. Furthermore, evidence suggests females are more likely than males to decline therapy for fear of side effects, or discontinue statin use due to intolerance [5, 6, 7]. Bempedoic acid is an adenosine triphosphate (ATP) citrate lyase inhibitor targeting cholesterol synthesis upstream of 3‐hydroxy‐3‐methylglutaryl coenzyme A reductase, the enzyme inhibited by statins [8]. Bempedoic acid is similar to statins in that it reduces hepatic cholesterol synthesis and raises LDL‐cholesterol receptor expression, thereby increasing the clearance of circulating LDL‐C. Bempedoic acid is a prodrug primarily activated in the liver by very long‐chain acyl‐CoA synthetase 1 (ACSVL1), and this enzyme is undetectable in skeletal muscle [8]. Thus, in contrast to statins, bempedoic acid may contribute to fewer adverse musculoskeletal effects.

There is great interest, particularly for females, in alternative LDL‐C lowering therapies demonstrating similar efficacy to statins in primary and secondary prevention but with improved adherence and tolerability. Females comprised from 24% to 29% of enrollees in recent cardiovascular outcomes studies of non‐statin lipid lowering therapies [9, 10, 11, 12]. Notably, CLEAR Outcomes included the highest percentage to date of females enrolled in a contemporary lipid lowering cardiovascular outcomes trial with 48.2% (*N* = 6740) [13]. The CLEAR Outcomes study demonstrated that compared to placebo, treatment with bempedoic acid was associated with a 13% lower risk of the primary endpoint of four component major adverse cardiovascular event (MACE‐4: cardiovascular death, nonfatal myocardial infarction, nonfatal stroke, and coronary revascularization) in statin intolerant patients at high cardiovascular risk [13]. As previously reported, there was no heterogeneity in cardiovascular risk reduction by sex (*p*‐value interaction 0.89) [14]. Leveraging the uniquely sizeable female cohort of CLEAR Outcomes, this analysis will explore the differences in baseline clinical characteristics and tolerability by sex with the goal of better informing the management of LDL‐C lowering in females.

## 2 Methods

CLEAR Outcomes was a double‐blind, randomized, placebo‐controlled trial which enrolled 13 970 patients from December 2016 to August 2019 from 1250 sites in 32 countries. Study participants were randomized to 180 mg oral bempedoic acid daily or placebo and followed for a median of 3.4 years. The full description of the study design and the primary outcomes of the trial have been published [13, 15]. Patients aged 18 to 85 years were eligible for enrollment if they had either sustained a previous cardiovascular event (secondary prevention) or were considered at high risk for a cardiovascular event (primary prevention) and had a baseline LDL‐C of at least 100 mg/dL (2.6 mmol/L). All patients were “statin intolerant” as defined in the protocol as being unwilling or unable to take guideline‐recommended doses of statins [15]. Concomitant treatment with a very low average daily statin dose (below the lowest approved dose) without side effects was permitted, as was administration of other stable lipid lowering therapies such as ezetimibe and proprotein convertase subtilisin‐kexin type 9 (PCSK9) inhibitors. Ethics committee approvals for the trial were obtained locally via relevant authorities or through a central institutional review board. Each patient provided written informed consent. Investigations were in accordance with the Declaration of Helsinki.

As pre‐specified CLEAR Outcomes trial efficacy data by sex have been previously published, here we describe post hoc the baseline clinical characteristics, tolerability, and discontinuation rates of enrolled participants by sex (11, 12). Classification of statin intolerance as statin‐associated muscle symptoms (SAMS) or non‐muscle related intolerance (non‐SAMS) at screening was by patient recall and or medical record charting. Tolerability data includes treatment‐emergent adverse events (AE), which were defined as events that began or worsened on or after the first dose of the study drug through study completion. Adverse events were coded to preferred terms and System Organ Class per the Medical Dictionary of Regulatory Activities (MedDRA), version 23.1. Laboratory abnormalities occurring after initiation of study drug are also reported.

### 2.1 Statistical Analysis

Baseline clinical characteristics are presented as frequencies (%) for categorical variables and means (standard deviation) or medians (interquartile range) for continuous variables in the intent to treat population. Treatment‐emergent AE were summarized in subject incidences. All comparisons were descriptive based on numerical differences only and no statistical testing was performed. Relative risks (RR) were calculated to compare treatment discontinuation rates. The treated population was analyzed for safety. Analyses were conducted with SAS, version 9.4 (Cary, NC).

#### 2.1.1 Role of the Funding Source

Funding for the CLEAR (Cholesterol Lowering via Bempedoic acid [ETC1002], an ACL‐Inhibiting Regimen) Outcomes study was provided by Esperion Therapeutics, Inc. The trial was designed by the sponsor in collaboration with the Cleveland Clinic Coordinating Center for Clinical Research (C5Research) and an academic executive committee. The sponsor reviewed this manuscript and provided suggested revisions, but the final decision on content was reserved for the academic authors with no restrictions on the right to publish.

## 3 Results

### 3.1 Baseline Characteristics of Female Versus Male Participants

**Figure 1:**
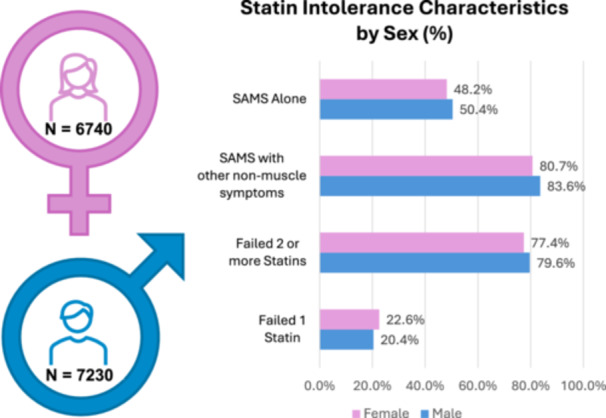
Baseline characterization of study entry criteria for statin intolerance by sex: SAMS ‐ statin associated muscle symptoms; Statin intolerance included any symptom of intolerance that started or increased during statin therapy and resolved or improved when statin therapy was discontinued, resulting in an inability to tolerate either 2 or more statins at any dose; or 1 statin at any dose and unwilling to attempt a second statin, or advised by a physician not to attempt a second statin.

Of the 13 970 patients randomized in CLEAR Outcomes, 6740 (48.1%) self‐identified as female. Females were older (mean age 66.8 years vs. 64.4 years for males), and although most patients enrolled self‐identified as white (Table 1), there were more females enrolled who identified as American Indian/Alaska Native and Black or African American compared to males (4.2% vs. 2.8% and 3.1% vs. 1.6%, respectively), while more males identified as Asian (2.5% vs. 1.2%). Additionally, more females identified as Hispanic or Latino (19.3% vs. 14.2%) compared to males. At baseline, females had a higher mean LDL‐C (144.4 mg/dL vs. 134.0 mg/dL), were more likely to have an elevated LDL ≥ 160 mg/dL (29.2% vs. 19%) and had a higher median high sensitivity C‐reactive protein (hsCRP, 2.6 mg/L vs. 2.0 mg/L) compared to males. Prior statin‐associated symptoms were characterized as statin‐associated muscle symptoms (SAMS) or non‐statin‐associated muscle symptoms (Non‐SAMS). Statin‐associated muscle symptoms alone accounted for almost half of the reasons for statin intolerance while patients experiencing both SAMS and Non‐SAMS accounted for more than 80% (Figure 1). Prior to enrollment, approximately 90% of females and males reported some impact of statins on daily living, although more females (14.7%) reported severe symptoms versus males (12%). Approximately 7% of males and females reported no change in daily living with statin use. Additionally, as enrollment criteria for statin intolerance, fewer females reported failing two or more statins than males, while fewer males reported failing only one statin at any dose (Figure 1). Among primary prevention patients there were more females (59.0%) than males (41.0%). Overall, 17.1% of females and 12.7% males had cerebrovascular atherosclerotic disease, 13.6% of females and 9.8% of males had peripheral arterial disease, and 38.4% of females and 62.6% of males had coronary artery disease. Females also reported more diabetes, hypertension, and chronic kidney disease than males at baseline (Table 1). While concomitant use of very low average daily statin dose as well as other lipid lowering therapies were permitted at enrollment, fewer females were on baseline statin therapy than males (20.5% and 24.8%, respectively) or other non‐statin lipid modifying treatments (16.8% female, 20.0% male). After initiation of the study, cross‐in lipid modifying therapy use for > 90 days occurred in 16.0% of males randomized to placebo and in 10.6% randomized to bempedoic acid; 15.2% of females randomized to placebo received cross‐in therapies, as did 8.2% randomized to bempedoic acid.

**Table 1:** Demographic and baseline patient characteristics by sex.

Select baseline characteristics	Female (*N* = 6740)	Male (*N* = 7230)
Age (years), mean (SD)	66.8 (8.4)	64.4 (9.4)
Age, years, *n* (%)		
< 65	2313 (34.3)	3453 (47.8)
> 65–< 75	3311 (49.1)	2786 (38.5)
> 75	1116 (16.6)	991 (13.7)
Race, *n* (%)		
White	6078 (90.2)	6654 (92.0)
Ethnicity, *n* (%)		
Hispanic or Latino	1304 (19.3)	1029 (14.2)
Body‐mass index (kg/m2), mean (SD)	30.1 (5.6)	29.7 (4.8)
LDL‐Cholesterol (mg/dL), mean (SD)	144.4 (36.6)	134.0 (32.7)
LDL‐Cholesterol category (mg/dL), *n* (%)		
< 130	2566 (38.1)	3597 (49.8)
≥ 130–< 160	2204 (32.7)	2259 (31.2)
≥ 160	1970 (29.2)	1374 (19.0)
hsCRP (mg/L), median (IQR)	2.6 (1.3–4.9)	2.0 (1.0–4.0)
hsCRP category (mg/L), *n* (%)		
< 2	2632 (39.4)	3509 (49.1)
> 2	4046 (60.6)	3641 (50.9)
HDL‐Cholesterol (mg/dL), mean (SD)	54.0 (13.8)	45.3 (11.3)
Non‐HDL‐Cholesterol (mg/dL), mean (SD)	179.0 (41.7)	169.1 (37.4)
Total Cholesterol (mg/dL), mean (SD)	233.0 (41.8)	214.5 (37.8)
Triglycerides (mg/dL), median (IQR)	159.0 (118.5–213.0)	159.5 (117.5–218.5)
Cardiovascular risk category, *n* (%)		
Primary prevention	2481 (36.8)	1725 (23.9)
Secondary prevention	4259 (63.2)	5505 (76.1)
Coronary artery disease	2586 (38.4)	4524 (62.6)
Peripheral artery disease	915 (13.6)	709 (9.8)
Cerebrovascular atherosclerotic disease	1151 (17.1)	916 (12.7)
Glycemic status, *n* (%)		
No diabetes	3411 (50.6)	4186 (57.9)
Normoglycemia	909 (13.5)	892 (12.3)
Pre‐Diabetes	2502 (37.1)	3294 (45.6)
Diabetes	3329 (49.4)	3044 (42.1)
Inadequately controlled diabetes	1407 (20.9)	1318 (18.2)
Hypertension, *n* (%)	5877 (87.2)	5999 (83.0)
eGFR category (mL/min/1.73 m2), *n* (%)		
≥ 90	1011 (15.0)	1438 (19.9)
60–< 90	4051 (60.1)	4553 (63.0)
30–< 60	1660 (24.6)	1221 (16.9)
< 30	17 (0.3)	18 (0.2)
Chronic kidney disease, *n* (%)	489 (7.3)	441 (6.1)
Baseline lipid‐modifying therapy use, *n* (%)	2514 (37.3)	3239 (44.8)
Baseline statin use, *n* (%)	1381 (20.5)	1793 (24.8)
Baseline ezetimibe use, *n* (%)	652 (9.7)	960 (13.3)

Abbreviations: CA, coronary artery disease; eGFR, estimated glomerular filtration rate; HDL, high density lipoprotein; hsCRP, high sensitivity *C*‐reactive protein; LDL, low density lipoprotein; PAD, peripheral artery disease.

### 3.2 Treatment Discontinuation and Adherence in Female Versus Male Participants

Of the 13 970 patients who underwent randomization, treatment discontinuation rates were 30.5% for bempedoic acid and 33.4% for placebo in females, while discontinuation occurred in 27.9% for bempedoic acid and 30.0% for placebo in males. Withdrawal of consent from the study was 2.7% in females and 2.4% in males. Overall treatment non‐adherence, defined as adherence to medical therapy < 80% of the time, occurred in 5.1% of females and 3.5% of males. A greater rate of non‐adherence was observed in females for both study arms (4.6% females and 2.9% males for bempedoic acid; 5.7% females and 4% males for placebo).

Non‐adherence did not appear to differ between males who had as their enrollment criteria for statin intolerance failed 1 statin at any dose versus those who had failed 2 or more statins at any dose (3% vs*.* 3.6%). For females, non‐adherence was slightly higher in those who had failed 2 or more statins versus those who had failed 1 statin at any dose (5.4% vs. 4.0%). The non‐adherence in those who had failed 2 or more statins versus those who failed 1 statin at any dose in the primary prevention cohort was 5.2% and 3.4%, respectively, of females; 3.3% and 2.9%, respectively, of males. Similar findings of non‐adherence were seen in the secondary prevention cohort for females who had failed 2 or more statins versus those who had failed 1 statin (5.6% and 4.5% of females and 3.6% and 3.1% of males, respectively).

### 3.3 Adverse Events in Female Versus Male Participants

The overall incidences of AEs, serious AEs, and AEs leading to discontinuation of study drug by sex are reported in Table 2. The incidences of investigator‐reported prespecified adverse events of special interest were similar between sexes. The incidence of myalgia was 5.4% and 5.9% for females and 5.8% and 7.6% for males treated with bempedoic acid and placebo, respectively. Discontinuation of study drug due to myalgia occurred in 1.8% and 1.6% of females, and 1.7% and 2.1% of males treated with bempedoic acid or placebo, respectively. As in the overall analysis, both males and females experienced a higher incidence of hepatic enzyme elevations, renal impairment, and hyperuricemia on bempedoic acid versus placebo [16]. In the bempedoic acid and placebo arms, repeated and confirmed aminotransferase elevations (ALT and AST level >3 times upper limit of normal (ULN)) are reported in Table 2. Renal impairment rates were 12.1% with bempedoic acid and 9.0% with placebo for females and 10.9% and 8.2%, respectively, for males. The mean (SD) change in creatinine at 6 months was 0.05 (0.16) mg/dL with bempedoic acid and 0.01 (0.14) mg/dL with placebo in females, and 0.05 (0.18) mg/dL with bempedoic acid and 0.01 (0.21) mg/dL with placebo in males. Prespecified renal AEs deemed of greater clinical importance were renal failure (1.2% bempedoic acid and 0.8% placebo for females, 1.0% bempedoic acid and 0.9% placebo for males) and acute kidney injury (0.9% bempedoic acid and 1.0% placebo in females, 1.3% bempedoic acid and 0.9% placebo in males). Hyperuricemia AE rates for bempedoic acid versus placebo were 10.2% and 5.5% for females, and 11.5% and 5.8% for males, respectively. Reporting of gout AE in females was approximately half that of males (Table 2). The mean (SD) change from baseline in uric acid level (mg/dL) at 6 months for bempedoic acid and placebo was 0.76 (1.15) and −0.01 (0.96) for females and 0.77 (1.28) and −0.04 (1.08) for males. Cholelithiasis was reported slightly more in females and for those on bempedoic acid. Additionally, neurocognitive disorders were reported similarly between females and males and those treated with bempedoic acid or placebo.

**Table 2:** Investigator‐reported adverse events in the safety population.

	Female		Male	
	Bempedoic acid (*N* = 3364)	Placebo (*N* = 3374)	Bempedoic acid (*N* = 3637)	Placebo (*N* = 3590)
Any adverse event	2933 (87.2%)	2859 (84.7%)	3107 (85.4%)	3060 (85.2%)
Any serious AE	814 (24.2%)	791 (23.4%)	953 (26.2%)	942 (26.2%)
AE leading to treatment discontinuation	388 (11.5%)	352 (10.4%)	371 (10.2%)	370 (10.3%)
Prespecified adverse events of special interest				
Myalgia	182 (5.4%)	198 (5.9%)	211 (5.8%)	273 (7.6%)
Myalgia leading to discontinuation	61 (1.8%)	54 (1.6%)	63 (1.7%)	75 (2.1%)
NODM (without diabetes at baseline)	238/1730 (13.8%)	263/1681 (15.6%)	383/2126 (18.0%)	377/2059 (18.3%)
(With Pre‐diabetes at baseline)	217/1255 (17.3%)	242/1247 (19.4%)	352/1663 (21.2%)	344/1630 (21.1%)
(With Normoglycemia at baseline)	21/475 (4.4%)	21/434 (4.8%)	31/463 (6.7%)	33/429 (7.7%)
Worsening hyperglycemia (with baseline diabetes)	355/1634 (21.7%)	354/1693 (20.9%)	358/1511 (23.7%)	392/1531 (25.6%)
Hypoglycemia	172 (5.1%)	140 (4.1%)	132 (3.6%)	127 (3.5%)
Acidosis	5 (0.1%)	7 (0.2%)	8 (0.2%)	4 (0.1%)
Elevated hepatic‐enzyme level	155 (4.6%)	97 (2.9%)	162 (4.5%)	112 (3.1%)
Renal impairment	406 (12.1%)	303 (9.0%)	396 (10.9%)	296 (8.2%)
Neurocognitive disorders	32 (1.0%)	37 (1.1%)	26 (0.7%)	32 (0.9%)
Atrial fibrillation	97 (2.9%)	100 (3.0%)	132 (3.6%)	146 (4.1%)
Adjudicated tendon rupture	31 (0.9%)	25 (0.7%)	54 (1.5%)	41 (1.1%)
Tendinopathies	60 (1.8%)	54 (1.6%)	58 (1.6%)	74 (2.1%)
Malignant conditions	123 (3.7%)	125 (3.7%)	198 (5.4%)	216 (6.0%)
Other Adverse Events				
Hyperuricemia	344 (10.2%)	185 (5.5%)	419 (11.5%)	208 (5.8%)
Gout	68 (2.0%)	43 (1.3%)	147 (4.0%)	100 (2.8%)
Cholelithiasis	91 (2.7%)	45 (1.3%)	61 (1.7%)	36 (1.0%)
Laboratory results at 6 months – mg/dL, mean (SD)				
Change from baseline in uric acid level	0.76 (1.15)	−0.01 (0.96)	0.77 (1.28)	−0.04 (1.08)
Change from baseline in creatinine level	0.05 (0.16)	0.01 (0.14)	0.05 (0.18)	0.01 (0.21)
Laboratory results after 12 months, mean (SD)				
Change from baseline in glycated hemoglobin level (%)	0.03 (0.78)	0.05 (0.67)	0.05 (0.71)	0.07 (0.73)
Abnormal enzyme level at any visit—no. (%)				
Creatine kinase level > 5× ULN, single occurrence	28 (0.8%)	20 (0.6%)	17 (0.5%)	20 (0.6%)
Creatine kinase level > 5× ULN, repeated and confirmed	5 (0.1%)	3 (0.1%)	3 (0.1%)	5 (0.1%)
Creatine kinase level > 10× ULN, single occurrence	10 (0.3%)	8 (0.2%)	8 (0.2%)	7 (0.2%)
Creatine kinase level > 10× ULN, repeated and confirmed	1 (< 0.1%)	3 (0.1%)	1 (< 0.1%)	1 (< 0.1%)
Alanine aminotransferase level > 3× ULN, repeated and confirmed	37 (1.1%)	23 (0.7%)	46 (1.3%)	30 (0.8%)
Aspartate aminotransferase level > 3× ULN, repeated and confirmed	38 (1.1%)	17 (0.5%)	42 (1.2%)	26 (0.7%)

Abbreviations: AE, adverse event; NODM, new onset diabetes mellitus; ULN, upper limit of normal.

## 4 Discussion

While disparities in care account for some component of sex differences with statin therapy [17], statin nonadherence appears to be more common amongst females than males. A recent study of patients at high risk for or with atherosclerotic disease showed nonacceptance of statin therapy as more common in females versus males (24.1% vs. 19.7%, *p* value < 0.001) [7]. Sex differences in statin adherence are complex but may, in part, be due to misinformation regarding the efficacy of lipid lowering therapy in females versus males, particularly for primary prevention. In the Patient and Provider Assessment of Lipid Management (PALM) registry [5], females were less likely than males to believe statins are safe and more likely to decline or discontinue a statin. It remains to be studied if these preconceived beliefs regarding efficacy of statins would affect real world adherence to bempedoic acid therapy. Like studies of statins, there was a trend in the CLEAR Outcomes trial towards higher nonadherence to bempedoic acid amongst females than males, although interestingly, the trends in both groups were higher for the placebo arm. These findings are similar to recent studies of statin intolerance showing a so‐called “nocebo” effect, wherein a negative perception of statins or lipid lowering therapies result in a negative outcome [18]. The rate of study drug discontinuation observed in this analysis ranged from 27.9%–33.4% depending on sex and treatment allocation. Approximately 2% of the study population discontinued study treatment due to the global COVID‐19 pandemic [19]. Prior studies in statin intolerant patients, which were notably of much shorter duration and in smaller populations, demonstrated 16%–25% study drug discontinuation rates [20, 21]. While the nocebo effect in a statin intolerant patient population might be considered a potential contributor to patients discontinuing study treatment, recent cardiovascular outcomes trials in statin tolerant patients have demonstrated discontinuation rates of 25%–30% in comparison [12, 22, 23]. Discontinuation rates in the placebo arm, given the higher rate of endpoint events, could also reflect patient decision to stop treatment due to a perceived lack of efficacy. It should be noted that withdrawal of consent from this study was comparable in males versus females, further suggesting that the sex differences in statin adherence may not be replicated with bempedoic acid.

Beyond efficacy, there is also the possibility of a greater incidence of statin‐associated side effects amongst females, and data suggest females are less likely than males to adhere to statin therapy due to intolerance [7, 24]. A meta‐analysis of more than 4 million patients reported that, while the overall prevalence of statin intolerance was 9.1% (95% CI: 8.0%–10%), females are 47.9% (OR: 1.47, *p* = 0.007) more likely to experience statin intolerance compared to their male counterparts. A slightly smaller proportion of females in the CLEAR Outcomes trial were on very low dose statin lipid lowering therapy, which patients were allowed to continue while participating, further suggesting potential sex differences in statin tolerability. Higher percentages of females enrolled in the study after failing only one statin and unwilling to try another. While the baseline Quality of Life (QOL) question for these patients suggested comparable reports of a negative impact of statins on QOL, women appeared to report a severe impact of statins on QOL. This does suggest there may be some validity to hypothesized sex differences in medication tolerability [17]. There was also a possibility that females intolerant to two or more statins were less likely to be adherent to bempedoic acid therapy, indicating that, for females, intolerance to other lipid lowering therapy may have informed willingness to continue with alternative therapy.

While the concept of statin intolerance remains controversial, alternative non‐statin lipid lowering therapies, given alone or in combination, are needed to manage patients to guideline recommended LDL‐C targets when the patient is unable or unwilling to take a statin. Of note, no difference was seen in the rate of adverse events in females versus males enrolled in CLEAR Outcomes. In the primary analysis of CLEAR Outcomes, the overall discontinuation rate was approximately 30% in both bempedoic acid and placebo arms and likely represents a multitude of contributing factors including treatment of a patient population who are unable and/or unwilling to take statins at baseline and participation in a clinical trial during a pandemic. Bempedoic acid was well‐tolerated in both males and females with lower rates of discontinuation within the bempedoic acid group compared to placebo group and similar rates of discontinuation between females and males. Data from the Understanding Statin Use in American and Gaps in Patient Education (USAGE) survey found women reported a statin being switched or stopped because of side effects, including new or worsening muscle symptoms (*p* < 0.01), more often than men [24]. In CLEAR Outcomes, the incidence of discontinuation of study drug due to myalgia was comparable between females and males in both arms, thus establishing bempedoic acid as a viable option in patients unable or unwilling to take statins due to muscle related symptoms. Furthermore, concerns surrounding neurocognitive disorders, often cited as reasons for statin refusal or discontinuation, was similar in the overall population and between males and females for bempedoic acid versus placebo. Disseminating this data to a broad range of health care providers, including internists as well as cardiologists, may be of particular importance given evidence suggesting more intensive LDL‐lowering among females receiving care from cardiologists [25]. This sub‐study of CLEAR Outcomes demonstrates that bempedoic acid is a viable alternative to statins, regardless of sex, and should be a considered within a practitioner's “tool belt” of lipid lowering options.

There are limitations to this sub‐study of CLEAR Outcomes. Although there is a need for studying lipid lowering therapies in patients with statin intolerance, restricted enrollment in CLEAR Outcomes to patients who had reported their inability and/or unwillingness to take statins may limit generalizability to the greater population of patients on guideline‐directed statin therapy. Additionally, the characteristics of males and females in this study as well as sex differences in adherence were not powered for statistical significance. The study was also not randomized by sex for bempedoic acid versus placebo, and therefore, sex differences observed would need validation in future studies randomized by sex. Nevertheless, these clinical characteristics and tolerability findings stratified by sex are clinically important, informing clinical practice as well as future trial recruitment.

## 5 Conclusions

CLEAR Outcomes provides insight into the characteristics of statin intolerance amongst females, a historically underrepresented population in randomized trials and one reported as having greater intolerance to statin therapy. Given findings that suggest bempedoic acid reduced MACE equally in females and males as compared to placebo, its comparable tolerability, and the impact of LDL‐lowering on reducing CV risk, bempedoic acid is an important additional tool for improving cardiovascular outcomes via LDL‐C lowering for primary and secondary prevention, particularly in patients who do not tolerate statins. Further research randomized by sex is needed to explore side effects and adverse events as well as adherence to bempedoic acid as compared to statin therapy.

## Author Contributions

Sahar Naderi, Leslie Cho were involved in the concept/design of the manuscript, data interpretation, and writing of the first draft of the manuscript. Danielle Brennan, Na Li, Paula Robinson, and Heather A. Powell were involved in the concept/design of the manuscript, data acquisition, formal analysis, and data interpretation. A. Michael Lincoff, Stephen J. Nicholls, Steven E. Nissen, Jorge Plutzky were involved in conceptualization, study methodology, supervision, and data interpretation. All authors critically reviewed the manuscript and approved the final version of the manuscript for submission.

## Conflicts of Interest

Na Li is an employee of Esperion Therapeutics, Inc. and may hold company stock or stock options as compensation. A. Michael Lincoff has received Esperion research funding for this trial; receiving grants from Eli Lilly, AbbVie, CSL, AstraZeneca, and Novartis; and received personal fees from Novo Nordisk, Glaxo, Akebia, Endologix, Fibrogen, Provention, and Becton Dickson. Stephen J. Nicholls has received research support from AstraZeneca, Amgen, Anthera, CSL Behring, Cerenis, Eli Lilly, Esperion, Resverlogix, Novartis, InfraReDx and Sanofi‐Regeneron and is a consultant for Amgen, Akcea, AstraZeneca, Boehringer Ingelheim, CSL Behring, Eli Lilly, Esperion, Kowa, Merck, Takeda, Pfizer, Sanofi‐Regeneron, Vaxxinity, CSL Sequiris and Novo Nordisk. Steven E. Nissen has received grant support from Esperion for the CLEAR Outcomes Trial. Cleveland Clinic Center for Clinical Research has received funding to perform clinical trials from Abbvie, AstraZeneca, Arrowhead, Amgen, Bristol Myers Squibb, Eli Lilly, Medtronic, MyoKardia, New Amsterdam Pharmaceuticals, Novartis, and Silence Therapeutics. Steven E. Nissen is involved in these clinical trials but receives no personal remuneration for his participation. Jorge Plutzky has reported Esperion, including steering committee for CLEAR Outcomes trial, Boehringer Ingelheim (grant support, clinical trial), Novo Nordisk (consultant, SELECT Steering Comm), New Amsterdam, Novartis (grant support), Myome (consultant), and Altimmune (consultant). Heather A. Powell is an employee of Esperion Therapeutics, Inc. and may hold company stock or stock options as compensation. Paula Robinson is an employee of Esperion Therapeutics, Inc. and may hold company stock or stock options as compensation. Leslie Cho reports serving on the steering committee for the CLEAR Outcomes trial. The other authors declare no conflicts of interest.

## Data Availability

Data used in the conduct of these analyses will not be made available to external parties.
